# Pilot Study on Genetic Associations With Age-Related Sarcopenia

**DOI:** 10.3389/fgene.2020.615238

**Published:** 2021-01-11

**Authors:** Felicita Urzi, Boštjan Pokorny, Elena Buzan

**Affiliations:** ^1^Faculty of Mathematics, Natural Sciences and Information Technologies, University of Primorska, Koper, Slovenia; ^2^Environmental Protection College, Velenje, Slovenia

**Keywords:** sarcopenia, MTHFR, ACTN3, NRF2, genetic factors

## Abstract

Despite strong evidence of an inheritable component of muscle phenotypes, little progress has been made in identifying the specific genetic factors involved in the development of sarcopenia. Even rarer are studies that focus on predicting the risk of sarcopenia based on a genetic risk score. In the present study, we tested the single and combined effect of seven candidate gene variants on the risk of sarcopenia. Single nucleotide polymorphisms in candidate genes were genotyped using the KASP assay. We examined 190 older adults that were classified as non-sarcopenic or sarcopenic according to the diagnostic criteria of the European Working Group on Sarcopenia in Older People. Sarcopenia was associated with Methylenetetrahydrofolate reductase, Alpha-actinin-3, and Nuclear respiratory factor 2 genotypes. The combined effect of all three polymorphisms explained 39% of the interindividual variation in sarcopenia risk. Our results suggest that the single and combined effect of Methylenetetrahydrofolate reductase, Alpha-actinin-3, and Nuclear respiratory factor 2 polymorphism is associated with sarcopenia risk in older adults. Nowadays, as the population is getting older and older, great efforts are being made to research the etiology, diagnosis and treatment of sarcopenia. At the same time, small progress has been made in understanding the genetic etiology of sarcopenia. Given the importance of research on this disease, further genetic studies are needed to better understand the genetic risk underlying sarcopenia. We believe that this small-scale study will help to demonstrate that there is still much to be discovered in this field.

## Introduction

The increasing prevalence of the elderly within the present population structure is the most prominent demographic aspect of the 21st century. The change in population structure constitutes a challenge to provide adequate healthcare and sustain social care systems worldwide (World Health Organization, 2015) since it is known that age-related changes in body composition lead to functional deterioration with negative health consequences. Sarcopenia, defined as “low muscle strength, low muscle quantity/quality, and low physical performance,” is one of the health conditions that older adults face (Morley et al., 2011; Cruz-Jentoft et al., 2019), and is classified as an age-related disease with an International Classification of disease (ICD-10CM) (M62.84) code (Anker et al., 2016). The consequences of sarcopenia are associated with loss of independence due to reduced physical capacity and mobility disorders that may lead to hospitalization and/or admission to a nursing home (Kerr et al., 2006; Landi et al., 2013; Correa-de-Araujo and Hadley, 2014). In most cases, this disease is associated with other chronic diseases and as such, is a significant burden on the public health system (Beaudart et al., 2014; Kim et al., 2014).

The etiology of sarcopenia is complex and can be attributed to a variety of factors, including oxidative stress, inflammation, apoptosis, and mitochondrial dysregulation (Roubenoff, 2003; Fulle et al., 2004), as well as genetic factors, inadequate diet, sedentary lifestyle, and the interplay between these factors (Rolland et al., 2008; Walrand et al., 2011). Although many aspects of sarcopenia are well understood, studies have only recently begun to investigate the genetic influences on skeletal muscle traits with studies estimating the heritability of muscle strength to be between 30–85% (Reed et al., 1991; Thomis et al., 1997; Huygens et al., 2004) and 50–80% (Bouchard et al., 1985; Seeman et al., 1996; Abney et al., 2001) for lean mass. In the field of sports genomics, a large number of genetic case-control studies have been published to evaluate candidate gene variants. Most studies have focused on the power/strength phenotype in elite athletes and found many genes linked to the muscle phenotypes (Ahmetov and Fedotovskaya, 2015). Genetic markers Methylenetetrahydrofolate reductase [MTHFR 1.p36.2 A > C (rs1801131)] and Alpha-actinin-3 [ACTN3 11.q13.2 C > T (rs1815739)] have shown positive associations with athlete status in three or more studies, while Nuclear respiratory factor 2 [NRF2 15q21.2 C > A (rs12594956)], Vitamin D receptor [VDR *Fok*I 12q13.1 T > C (rs2228570)], Adrenoceptor beta 2 [ADRB2 5q32 G > A (rs1042713)], and Neuronal PAS domain-containing protein 4 [NPAS4 11q13.2 A > G (rs7947391)] in two or more studies, thus providing evidence for potentially important DNA polymorphisms that contribute to muscle-related phenotypes (Ahmetov and Fedotovskaya, 2015). Some candidate genes (e.g., angiotensin I converting enzyme, Alpha-actinin-3, thyrotropin releasing hormone receptor, vitamin D receptor, Nuclear respiratory factor 2, and Methylenetetrahydrofolate reductase) have also been studied in association with skeletal muscle mass and strength phenotypes in older adults (Roth, 2012). Roth et al. (2004) reported significant associations with the VDR (rs2228570) polymorphism and leg fat free mass in older men. The rs2228570 variant was also reported to be associated with muscle strength in numerous studies (Roth, 2012). The polymorphism in the ACTN3 (rs1815739) showed a modest association with skeletal muscle strength and power in a number of studies (Clarkson et al., 2005; Vincent et al., 2007; Walsh et al., 2008), but the work of Delmonico et al. (2007) and Judson et al. (2011) pointed to the potential clinical importance of this variant in older men and women. Furthermore, the ACTN3 577X allele was associated with decrease in physical performance and an increase in frailty in a cohort of older individuals (Ma et al., 2018). There is supporting evidence that NRF2 contributes to the maintenance of muscle mass and function by regulating mitochondrial biogenesis and dynamics (Always et al., 2017). Certain results indicate that NRF2 deficiency aggravates frailty and sarcopenia during aging, at least in part by reducing the expression levels of genes related to oxidative stress and mitochondrial biogenesis, by reducing mitochondrial number, mitochondrial content, mtDNA copy number, and impaired mitochondrial morphology in skeletal muscle (Ibebunjo et al., 2013; El Assar et al., 2017; Huang et al., 2019). Studies have also highlighted the relevance of MTHFR polymorphism with variation in lean body mass (Liu et al., 2008) and possible manifestation of frailty (Ma et al., 2020). Elevated plasma homocysteine, due to MTHFR functional variant, has been suggested as a biomarker of physical function, such as physical limitation (Swart et al., 2013), reduced walking speed (Vidoni et al., 2017), and grip strength (Swart et al., 2013; Vidoni et al., 2018).

Despite strong evidence of a heritable component of muscle phenotypes (Carmelli et al., 2000; Zhai et al., 2005), little progress has been made in identifying the specific genetic influences on sarcopenia. To date, studies focusing on genes associated with sarcopenia itself are rare; only the influences of the genetic variants of Alpha-actinin-3 (Cho et al., 2017), Caveolin-1 (Lin et al., 2014) and vitamin D receptor (Roth et al., 2004) were investigated in connection with sarcopenia so far. Furthermore, none of the previous studies included genetic information to predict the risk of sarcopenia based on a genetic risk score with multiple single nucleotide polymorphisms (SNPs).

Interpreting the effects of genetic factors on individual variations in muscle strength, quality and functionality would be important to better understand the development of this disease. In this study, we investigated the importance of genetic information in predicting the risk of sarcopenia. We examined whether the individual polymorphism in the Methylenetetrahydrofolate reductase [MTHFR 1.p36.2 A > C (rs1801131)], Alpha-actinin-3 [ACTN3 11.q13.2 C > T (rs1815739)], Nuclear respiratory factor 2 [NRF2 15q21.2 C > A (rs12594956)], Vitamin D receptor [VDR *Fok*I 12q13.1 T > C (rs2228570)], Adrenoceptor beta 2 [ADRB2 5q32 G > A (rs1042713)], C-X3-C motif chemokine receptor 1 [CX3CR1 3.p22 C > T (rs3732379)], and Neuronal PAS domain-containing protein 4 [NPAS4 11q13.2 A > G (rs7947391)] genes are associated with sarcopenia in older adults. In addition, we tested the combined contribution of the relevant polymorphism by calculating the Total Sarcopenia Genetic Risk Score (SGS).

## Materials and Methods

### Participants

From March to July 2019, we invited older adults living in nursing homes in the south of Slovenia to participate in our study. Written consent was obtained from 190 eligible residents; all of whom met the inclusion criteria. Selection criteria included both men (*n* = 67) and women (*n* = 123) aged 65 years or older (average age 78.3 ± 9.6 years) with no difficulties in performing basic daily activities; no active treatment of cancer; no hyperthyroidism, hyperparathyroidism, chronic renal failure, rheumatoid arthritis, or heart disease. We have conducted our study in accordance with the principles of the Helsinki Declaration. The study was approved by the Republic of Slovenia National Medical Ethics Committee (0120-360-2018-10).

#### Population Stratification

Samples were collected from the same population without differences between the case and control groups. All participants were Caucasian, in the same age group and living in nursing homes. Therefore, we excluded population stratification as a potential source of confounding on the genotypic distribution and the effect on disease risk.

### Research Design

To validate the gene polymorphisms, we divided our participants into two groups: sarcopenic and non-sarcopenic.

Sarcopenia was evaluated according to the diagnostic criteria of the European Working Group on Sarcopenia in Older People (EWGSOP) in a cross-sectional study in nursing homes. At the beginning of the investigation, we carried out anthropometric, physical performance and strength measurements.

Body mass was measured with a calibrated digital scale, with participants standing barefoot and wearing light clothing. Body height was measured with a standard stadiometer attached to a wall without a footer. The body composition was measured by Bioelectrical impedance analysis (BIA; Maltron Bioscan 920, Rayleigh, United Kingdom, 50 kHz). Fat free mass (kg) and fat mass (kg) were derived from the instrument. The total skeletal muscle mass of the body was calculated using the equation: skeletal muscle mass (kg) = {(body height^2^/BIA resistance ⋅ 0.401) + (sex ⋅ 3.825) + [age ⋅ (–0.071)]} + 5.102 (Janssen et al., 2000). The skeletal muscle index (SMI) was calculated as muscle mass (kg) divided by the square of height (m). Physical performance was examined by usual gait speed over a 4 m course. Strength was measured as grip strength with a Handgrip Dynamometer (Jamar Hydraulic Hand Dynamometer, Homecraft Ltd., Nottinghamshire, United Kingdom), and was defined as the best performance of two attempts by the dominant hand.

#### Assessment of Sarcopenia (EWGSOP Algorithm)

The diagnosis of sarcopenia was based on the EWGSOP algorithm (Cruz-Jentoft et al., 2010, 2019). In the present study, we adopted the cut-off points indicated in the EWGSOP consensus paper. The cut-off point of gait speed of <0.8 m/s indicates participants with low physical performance. Low muscle strength was classified as hand grip strength <27 kg for men, and <16 kg for women. Low muscle mass was classified as a skeletal muscle index of less than 8.87 and 6.42 kg/m^2^ for men and women, respectively (Cruz-Jentoft et al., 2010, 2019).

#### SNP Selection

Single nucleotide polymorphisms were selected by searching existing databases^1^ ^,2^ ^,3^. The following criteria were used to identify the SNPs: (a) preliminary data for association with sarcopenia (ACTN3, VDR) or elite athletics status in previous publications (MTHFR, ADRB2); (b) genes related to cellular toxicity and protein degradation (MTHFR, CX3CR1), mitochondrial (NRF2) or neurological (NPAS4) regulation, muscle protein function (ACTN3), and the hormonal system (ADRB2) as contributing factors in the etiology of sarcopenia, and (c) a minor allele frequency of ≥0.1.

#### Sample Collection and Genotyping

Saliva samples were collected in the DNA stabilization buffer. The genomic DNA was extracted from saliva using the DNA| OG-500 Kit (DNA Genotek Inc.) following the manufacturer protocol. The DNA quality was evaluated by NanoDrop spectrometry and quantified with the Qubit dsDNA Broad Range Assay Kit (Invitrogen, Q32853). All DNA samples were stored at −80°C. Polymorphism of the MTHFR 1.p36.2 A > C (rs1801131), ACTN3 11.q13.2 C > T (rs1815739), NRF2 15q21.2 C > A (rs12594956), VDR *Fok*I 12q13.1 T > C (rs2228570), ADRB2 5q32 G > A (rs1042713), CX3CR1 3.p22 C > T (rs3732379), and NPAS4 11q13.2 A > G (rs7947391) genes were genotyped using KASP assay based on competitive allele-specific PCR (polymerase chain reaction), according to the manufacturer protocol (LGC, Biosearch Technologies, United Kingdom). The PCR reaction was performed with a final volume of 10 μL per reaction [5 μL genomic DNA (10 ng), 5 μL KASP Master mix (2x), 0.14 μL KASP assay mix]. The thermal cycle protocol for KASP genotyping reactions consisted of Step one: 1 cycle of activation at 94°C for 15 min; Step two: 10 cycles of denaturation at 94°C for 20 s, and annealing/elongation at 61–55°C (drop 0.6°C per cycle) for 60 s; Step three: 26 cycles of denaturation at 94°C for 20 s, and annealing/elongation at 55°C for 60 s. The protocol was performed with the LightCycler 96 Real-Time PCR System (Roche Molecular Systems, Inc., Pleasanton, CA, United States).

Polymorphisms of the candidate genes were genotyped using a standardized genotyping technique (KASP assay), based on competitive allele-specific PCR. The KASP assay exhibited a 93.5% amplification rate and allele call quality of 98%. For samples with missing genotype results, the DNA extraction and genotyping were repeated. All analyses were performed using the negative control. We repeated the genotyping in a randomly chosen sample (10%), using the same technique to ensure genotyping accuracy.

#### Calculation of Total Sarcopenia Genetic Risk Score

Only the gene variants associated with sarcopenia were included in the calculation of the total sarcopenia genetic risk score. To quantify the combined contribution of polymorphisms associated with sarcopenia, we used an algorithm resulting from the accumulation of genotype scores for each individual (Williams and Folland, 2008). The polygenic profile was calculated assuming an additive effect, with all three gene variants given equal weight in the total score. Specifically, each polymorphism genotype was evaluated with a genetic score based on the association with an unfavorable phenotype (i.e., sarcopenia status). The “unfavorable” homozygous genotype was rated 2, the heterozygous genotype 1, and the non-risk homozygous genotype 0. The total SGS was then mathematically converted to a scale from 0 to 100% (where an SGS of 100% represents a “perfect” polygenic profile for sarcopenia, and an SGS of 0% represents the “worst” profile for sarcopenia). Taking into account the genotype scores of three genes, the total sarcopenia genetic risk scores were calculated according to the equation:

SGS = (100/6) × (genotype scores MTHFR + genotype scores ACTN3 + genotype scores NRF2).

### Statistical Analysis

Data were analyzed using SPSS version 25 (SPSS Inc., Chicago, IL, United States). The χ2 was used to determine whether the populations were in Hardy-Weinberg equilibrium (HWE).

All analyses were performed separately for each individual SNP. One-way analysis of variance (ANOVA) followed by the Tukey’s *post hoc* test were used to check for differences in physical characteristics across genotype groups. In the ANOVA tests, the results for men and women were analyzed separately.

Investigations of genetic associations with sarcopenia were carried out with two tests. Firstly, χ2 was used to test the association of case-control genotype or allele counts. Secondly, associations with sarcopenia were examined by binary logistic regressions, with a correction for measured covariates (age, sex, and physical activity). Dominant, recessive, and SGS models were also tested. Each result was expressed as an odds ratio (OR) and 95% confidence interval (95% CI) with reference to the control group. The cut-off point for the SGS model was calculated with the receiver operating characteristic curve and Yuden index. All statistical tests were two-sided. A *p*-value of <0.05 was considered statistically significant.

Sample power was calculated using a Genetic power calculator^4^. The case-control parameters were set to: number of cases = 50, number of controls = 150, high risk allele frequency (A) = 0.4, prevalence = 0.25, genotypic relative risk Aa = 1.5, genotypic relative risk AA = 2.5, alpha = 0.05, to reach 80% power.

## Results

The classification of sarcopenia according to the algorithm proposed by EWGSOP resulted in a proportion of *n* = 45 (23.7%) of sarcopenic participants, and *n* = 145 (76.3%) of the control group. No significant differences in mean age (*p* = 0.080), height (*p* = 0.337), weight (*p* = 0.178), body mass index (*p* = 0.070), or body fat percentage (*p* = 0.231) were observed between the groups.

### Hardy-Weinberg

The genotype frequencies for MTHFR (AA = 44.5%, AC = 41.4%, CC = 13.6%; *p* = 0.430), ACTN3 (RR = 30.0%, RX = 46.8%, XX = 23.2%; *p* = 0.550), NRF2 (AA = 20.5%, AC = 55.3%, CC = 24.2%; *p* = 0.282), VDR *Fok*I (CC = 37.2%, CT = 49.7%, TT = 12.6%; *p* = 0.523), ADRB2 (GG = 41.1%, AG = 41.1%, AA = 17.9%; *p* = 0.185), and NPAS4 (AA = 9.9%, AG = 55.5%, GG = 34.0%; *p* = 0.063) were in Hardy-Weinberg equilibrium in the total studied samples. Genotype frequencies for the CX3CR1 polymorphism (CC = 49.7%, CT = 33%, TT = 16.8%; *p* = 0.011) deviated from the expected Hardy-Weinberg equilibrium and so we excluded the CX3CR1 gene from all further analysis. In the control samples, all five gene polymorphisms tested were in Hardy-Weinberg equilibrium. The deviation from the Hardy-Weinberg equilibrium was observed only in the sarcopenic samples (cases) for the MTHFR polymorphism (χ2 = 10.803, *p* = 0.001). The results of our study demonstrate a deficiency of homozygotes in the sarcopenic group.

### The Chi-Square (χ2) Association Test

**FIGURE 1:**
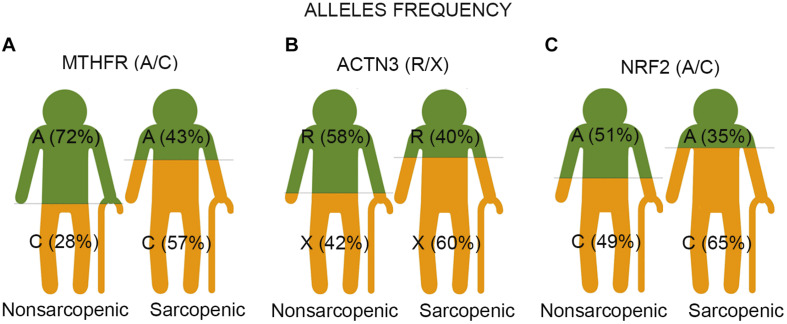
The representative images show the result of three single nucleotide polymorphisms (SNPs) associated with sarcopenia in older adults. Each image represents two groups of participants – non-sarcopenic and sarcopenic. **(A)** The study identifies the C allele of polymorphic variants (rs1801131 A/C) of the MTHFR gene as a risk factor for sarcopenia in older adults. The Chi-square association test showed significantly higher frequencies of the C allele in sarcopenic participants than in controls (57% vs. 28%, *p* < 0.001). The C-allele carriers had 3.3 increased odds ratios for the likelihood of being sarcopenic compared to the A-allele carriers. **(B)** The X allele of ACTN3 R577X (rs1815739 R/X) was associated with sarcopenia risk in older adults. The Chi-square association test showed significantly higher frequencies of the X allele in sarcopenic participants than in controls (60.0 vs. 42%, *p* = 0.003). The X allele carriers had 2.0 increased odds ratios for the likelihood of being sarcopenic compared to R allele carriers. **(C)** The C allele of NRF2 (rs12594956 A/C) was identified as a risk factor for sarcopenia in older adults. The Chi-square association test demonstrated significantly higher frequencies of the C allele in sarcopenic participants than in controls (65 vs. 49%, *p* = 0.009). The C-allele carriers had 1.9 increased odds ratios for the likelihood of being sarcopenic compared to carriers of the A allele.

The χ2 association test, used to compare genotype count, showed significant differences between sarcopenic older adults and controls for the genes MTHFR, ACTN3, and NRF2. Sarcopenia was associated with the genotype distribution of the MTHFR in rs1801131 (*p* < 0.001), ACTN3 in rs1815739 (*p* = 0.043), and NRF2 in rs12594956 (*p* = 0.015) polymorphisms. The frequencies of the C allele of the rs1801131 polymorphism in the MTHFR gene (56.7 vs. 28.3%, OR = 3.317, 95% CI = 2.034–5.409, *p* < 0.001), of the X allele of the rs1815739 polymorphism in the ACTN3 gene (60.0 vs. 42.4%, OR = 2.037, 95% CI = 1.258–3.297, *p* = 0.003), and of the C allele of the rs12594956 polymorphism in the NRF2 gene (64.4 vs. 48.6%, OR = 1.915, 95% CI = 1.174–3.124, *p* = 0.009) were significantly higher in sarcopenic participants than in controls (Figure 1). There was no association between genotype distribution and allele frequencies in the polymorphism of the ADRB2 (rs1042713), VDR *Fok*I (rs2228570), and NPAS4 (rs7947391) genes with sarcopenia (Supplementary Table 1).

### Binary Logistic Regression Analyses

Binary logistic regression analyses (unadjusted model) showed that sarcopenia was associated with MTHFR (AC + CC; OR = 2.428, 95% CI = 1.179–4.999, *p* = 0.016), with ACTN3 (RX + XX; OR = 2.753, 95% CI = 1.145–6.618, *p* = 0.024) and with NRF2 (AC + CC; OR = 3.261, 95% CI = 1.091–9.748, *p* = 0.034) under a dominant model, and with MTHFR (CC; OR = 11.043, 95% CI = 4.502–27.085, *p* < 0.001), with ACTN3 (XX; OR = 2.305, 95% CI = 1.104–4.816, *p* = 0.026), and with NRF2 (CC; OR = 2.429, 95% CI = 1.174–5.025, *p* = 0.017) under a recessive model, respectively (Table 1). Binary logistic regression analyses with included additional covariates (age, sex, and physical activity) highlighted that with advancing age, sarcopenia was associated with MTHFR (AC + CC; OR = 2.645, 95% CI = 1.221–5.730, *p* = 0.014) and with ACTN3 (RX + XX; OR = 2.531, 95% CI = 1.018–6.291, *p* = 0.046) under a dominant model, and with MTHFR (CC; OR = 13.513, 95% CI = 4.8862–37.373, *p* < 0.001), with ACTN3 (XX; OR = 2.248, 95% CI = 1.020–4.957, *p* = 0.045), and with NRF2 (CC; OR = 2.196, 95% CI = 1.014–4.758, *p* = 0.046) under a recessive model (Table 1). Under the dominant model, the contribution to the interindividual variability in sarcopenia trait was 4.8% for the MTHFR gene, 5% for the ACTN3 gene, and 4.4% for the NRF2 gene. For the recessive model, the contribution of the MTHFR gene to sarcopenia risk was 22%, the ACTN3 gene 3.7%, and the NRF2 gene 4.3%, respectively.

**TABLE 1:** Odds ratio (OR) and 95% confidence interval of MTHFR, ACTN3, and NRF2 genotypes for sarcopenia.

	Unadjusted models				Adjusted models			
		
	Dominant		Recessive		Dominant		Recessive	
				
	*p*	*OR* (95% CI)	*p*	*OR* (95% CI)	*p*	*OR* (95% CI)	*p*	*OR* (95% CI)
								
		AA/AC + CC		AA + AC/CC		AA/AC + CC		AA + AC/CC
MTHFR	0.016	2.428 (1.179–4.999)	<0.001	11.043 (4.502–27.085)	0.014	2.645 (1.221–5.730)	<0.001	13.513 (4.8862–37.373)
		RR/RX + XX		RR + RX/XX		RR/RX + XX		RR + RX/XX
ACTN3	0.019	2.857 (1.190–6.860)	0.026	2.305 (1.104–4.816)	0.046	2.531 (1.018–6.291)	0.045	2.248 (1.020–4.957)
		AA/AC + CC		AA + AC/CC		AA/AC + CC		AA + AC/CC
NRF2	0.034	3.261 (1.091–9.748)	0.017	2.429 (1.174–5.025)	0.082	2.765 (0.878–8.705)	0.046	2.196 (1.014–4.758)

*Binary logistic analyses were used to estimate the odds ratio (OR) and 95% confidence interval (CI) of genotypes related to sarcopenia. Model is adjusted for age, sex, and physical activity level. Dominant: AA versus AB + BB; recessive AA + AB versus BB (allele B increases risk).*

### Sarcopenia Genetic Risk Score

**FIGURE 2:**
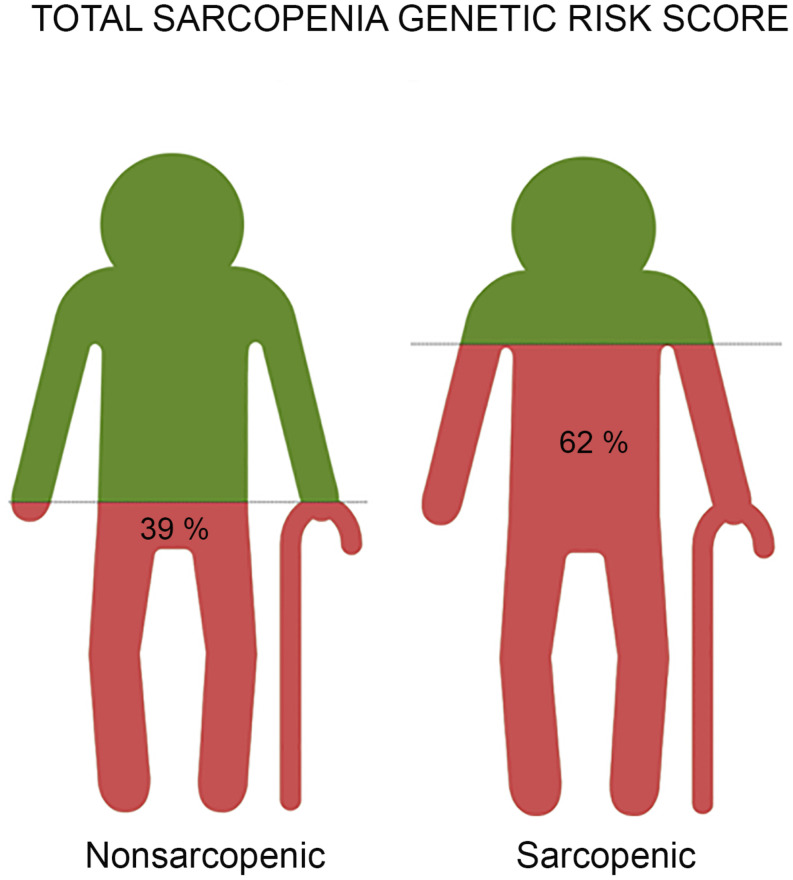
The representative images reveal the result of risk prediction based on a total genetic risk score for sarcopenia with the combined effect of all three (MTHFR, ACTN3, and NRF2) gene polymorphisms associated with sarcopenia in older adults. Each image represents a group of participants - non-sarcopenic and sarcopenic. We found a significantly higher total genetic risk score in sarcopenic participants compared to controls (62% vs. 39%, *p* < 0.001). Older adults with a genetic risk score of ≥58.3% had 1.9 increased odds ratios for the likelihood of being sarcopenic compared to those with a lower total sarcopenia genetic risk score.

To assess the combined effect of all three (MTHFR, ACTN3, and NRF2) genetic polymorphisms, the total sarcopenia genotype risk score was calculated. The proportion of participants with a higher genetic risk score for sarcopenia was higher in sarcopenic participants than for those in the control group (61.4% vs. 39.7%). An increased risk was found in participants with a higher total sarcopenia genetic risk score (OR = 1.057, 95% CI = 1.034–1.080, *p* < 0.001) (Figure 2). The total sarcopenia genetic risk score model predicted 39% of the variance in sarcopenia risk across the groups. The cut off point for the total sarcopenia genetic risk score model in our sample was 58.3% (*p* < 0.001; AUC = 0.774; 95% CI = 0.695–0.853) (Supplementary Tables 3, 4).

### Physical Characteristics According to the Given Genotypes

The physical characteristics according to MTHFR, ACTN3, NRF2, ADRB2, VDR *Fok*I, and NPAS4 genotypes are presented in Supplementary Table 2. In women, significant differences in the MTHFR genotypes were found with respect to skeletal muscle mass index (*p* = 0.019) and grip strength (*p* = 0.024). Tukey’s *post hoc* tests showed lower skeletal muscle mass index for CC homozygotes compared to AC heterozygotes (*M* = 0.65 kg/m^2^, *p* = 0.021) and AA homozygotes (*M* = 0.67 kg/m^2^, *p* = 0.021). Similarly, the results in women showed a lower grip strength for CC homozygotes compared to AA homozygotes (*M* = 4.9 kg, *p* = 0.018). In men, differences were observed in the MTHFR genotypes, with lower grip strength in CC homozygotes compared to AC heterozygotes (*M* = 6.81 kg, *p* = 0.048). In addition, the results in men showed significant differences in the NRF2 genotypes in grip strength (*p* = 0.015) and usual gait speed (*p* = 0.024). The results showed lower grip strength for CC homozygotes compared to AC heterozygotes (*M* = 9.8 kg, *p* = 0.028), and for CC homozygotes compared to AA homozygotes (*M* = 12.1 kg, *p* = 0.014). Differences in usual gait speed (*M* = 0.4 m/s, *p* = 0.025) were also noted in CC homozygotes compared to AA homozygotes. A borderline non-significant difference (*p* = 0.058) for grip strength was recorded in the NRF2 genotypes in women (with CC homozygotes having a lower strength than AA and AC carriers), and in ACTN3 genotypes in men (*p* = 0.054), with XX homozygotes having a lower strength than RR and RX carriers. No differences in physical characteristics were observed in ADRB2, VDR *Fok*I, and NPAS4 genotypes.

## Discussion

Sarcopenia is a geriatric syndrome that involves loss of muscle mass, strength and quality, and leads to the loss of function and independence in older adults. Previous studies have shown that genetic factors play an important role in the regulation of muscle characteristics and the sarcopenia phenotype (Delmonico et al., 2007; Liu et al., 2008; Walsh et al., 2016). However, they focused primarily on identifying the individual genes in order to understand the genetic susceptibility to this disease. Since each of these genes has only a small to moderate effect, it is important to use a polygenic model to explain the genetic variations in sarcopenia risk.

Therefore, we focused on the determination of the single and combined effect of MTHFR, ACTN3, NRF2, VDR *Fok*I, ADRB2, and NPAS4 polymorphism associated with sarcopenia in older adults. Our study has shown an association between polymorphisms in the genes MTHFR, ACTN3, and NRF2 and sarcopenia. As far as we know, this study highlighted for the first time the association of MTHFR and NRF2 polymorphisms and sarcopenia. Although no significant differences in sarcopenia risk were observed in ADRB2, and NPAS4 genotypes, evidence points to the importance of these genes in muscle strength (Ahmetov and Fedotovskaya, 2015; Rankinen et al., 2016) and quality (Kharraz et al., 2013; Arnold et al., 2015; Chazaud, 2016). No association with the VDR *Fok*I polymorphism was detected.

### Single Gene Polymorphism Associated With Sarcopenia in Older Adults

Our results have shown a genetic association of the MTHFR gene with sarcopenia. We found that the genotype and allelic frequencies of the MTHFR gene differed significantly between the sarcopenic participants and the control group. Under the dominant model, carriers of the MTHFR C allele had 2.6-fold, and under recessive models, the MTHFR C homozygotes had a more than 12-fold increased risk of sarcopenia with increasing age, even after adjustments for age, sex, and physical activity. The results suggest that the MTHFR C allele may contribute to interindividual variations in muscle traits and sarcopenia risk. The association with the physical characteristics of the participants showed that among women, MTHFR C carriers have a greater risk of low grip than A carriers. Besides, C carriers also have a greater risk of low skeletal muscle mass.

The C allele of the polymorphic variant A1298C (rs1801131) of the MTHFR gene, where the adenine to cytosine transversion changes glutamate into an alanine residue, is associated with reduced MTHFR enzyme activity, and elevated homocysteine levels due to the lack of 5-methyltetrahydrofolate, the necessary methyl donor for the conversion of homocysteine to methionine (Picciano et al., 1990). There is evidence that elevated homocysteine levels cause immune activation and cellular toxicity, possibly through Endoplasmic Reticulum (ER) stress, activation of the unfolded protein response and increased protein degradation (Jakubowski, 2006).

The relation of MTHFR 1298C polymorphism with elevated homocysteine has been consistently confirmed in different studies (Weisberg et al., 1998; Friedman et al., 1999; Ma et al., 2020). Studies show that elevated homocysteine levels are associated with both reduced physical performance (Kado et al., 2002; Kuo et al., 2007; Wong et al., 2013; Álvarez-Sánchez et al., 2020; Ma et al., 2020) and reduced muscle strength in older adults (Kado et al., 2002; Kuo et al., 2007). Previous findings have suggested that MTHFR gene variants are associated with lean body mass and may play an important role in the interindividual variation of these traits (Liu et al., 2008). However, some studies found elevated plasma homocysteine concentrations associated with physical performance and reduced muscle strength only in older women (Swart et al., 2013; Vidoni et al., 2018). In the present study, the MTHFR C allele was associated with significant reduction in muscle mass and strength in female carriers only. The reason for the lack of a significant association in men could be due to the gender-specific handling of the methionine signaling pathway, e.g., the rate of remethylation of homocysteine in the MTHFR pathway, which was seen to be significantly higher in women than in men (Fukagawa et al., 2000). Therefore, this could make women more susceptible to non-functional variants in this gene.

Our work suggests that the NRF2 gene may contribute to susceptibility to sarcopenia. The NRF2 C allele was overrepresented in the sarcopenic participants compared to the controls. In addition, sarcopenia was associated with the NRF2 polymorphism in both the dominant and recessive unadjusted regression models. The results of the adjusted regression model revealed a twofold higher risk of sarcopenia in CC homozygotes compared to A allele carriers. Furthermore, there was a greater risk of low grip and low gait speed in men NRF2 C carriers. Among women, NRF2 C carriers showed a similar trend.

The NRF2 gene is expressed as four distinct isoforms that result from alternative mRNA splicing (Gugneja et al., 1995). The intronic SNP NRF2 (C > A, rs12594956) may influence the expression of the alternative spliced transcripts of the gene, presumably resulting in decreased NRF2-dependent gene transcription. Binding sites for the NRF2 protein have been identified in several nuclear genes, including respiratory genes, genes for heme biosynthesis, and mitochondrial protein import (Gleyzer et al., 2005), in addition to mitochondrial DNA transcription, translation and replication (Garesse and Vallejo, 2001). Suppressed NRF2 signaling is often present in many pathological conditions, such as sarcopenia. Indeed, mitochondrial deterioration of muscles and motor neurons are recognized as the primary triggers of sarcopenia (Always et al., 2017). There is evidence that lower expression of NRF2 in skeletal muscle is associated with a higher risk of frail phenotype and impaired physical function in older adults (El Assar et al., 2017). Animal-related studies have shown that NRF2 deficiency affects physical function and muscle mass mainly in an age-dependent manner (Huang et al., 2019). The results demonstrate that nuclear encoded mitochondrial genes regulated by NRF2 were downregulated and coincide with the onset and progression of sarcopenia, providing the molecular signatures for the decline in oxidative capacity of muscle fibers (Ibebunjo et al., 2013). In this regard, studies suggest that both muscle mass and muscle strength - phenotypes that characterize sarcopenia - play a role in the age-related loss of aerobic capacity (de Oliveira et al., 2009). Moreover, it was confirmed that polymorphisms of the NRF2 gene (C > A, rs12594956) affect elite endurance performance, where the A allele is a positive predictor of the athletic endurance capacity (Eynon et al., 2013; Ahmetov and Fedotovskaya, 2015; Peplonska et al., 2017).

The current study, which focuses on sarcopenia itself, supports the earlier finding by Cho et al. (2017), that the ACTN3 genotype is significantly associated with sarcopenia in older adults. Under the adjusted recessive model, the ACTN3 X homozygotes showed a more than twofold increased risk of sarcopenia with increasing age. Despite the strong association with sarcopenia, no single muscle characteristic was associated with the ACTN3 polymorphism, except for non-significant differences in grip strength for ACTN3 XX homozygotes in men.

The ACTN3 gene has gained considerable attention following a number of cross-sectional studies in elite athletes that show some disadvantage for homozygote carriers of the R577X nonsense (X) allele in power performance events (Ahmetov and Fedotovskaya, 2015).

The ACTN3 gene encodes the α-actinin-3 protein, expressed only in fast type II muscle fibers and functions as a structural component of the sarcomere Z line. An allelic polymorphism in this gene (R577X, rs1815739), where a C-to-T base substitution results in the transformation of an arginine base (R) into a premature stop codon (X) causing the absence of the alpha-actinin-3 protein in the muscle fibers, can lead to loss of muscle function and weaken muscle strength/power (North et al., 1999). X allele homozygotes are deficient in the alpha-actinin-3 protein, which is associated with a lower fast-twitch fiber percentage (MacArthur and North, 2007).

Studies investigating the relationship between muscle strength and ACTN3 polymorphism in older adults are controversial. Some exhibited lower muscle strength in XX vs. R-allele carriers (Clarkson et al., 2005; Vincent et al., 2007; Walsh et al., 2008) while others did not support this association (Delmonico et al., 2007; McCauley et al., 2009; Norman et al., 2009; Hanson et al., 2010). Different studies reported an association between muscle mass and the ACTN3 X allele (Walsh et al., 2008; Zempo et al., 2010; Cho et al., 2017), but their findings were not confirmed by other studies (Delmonico et al., 2007; Vincent et al., 2007; Norman et al., 2009). Additionally, Judson et al. (2011) found that women carrying the X allele had a greater risk of falling than RR homozygotes.

In view of the varying results, the absence of a disease phenotype secondary to α-actinin-3 deficiency could arise due to compensation by the homologous protein, α-actinin-2. However, the high degree of evolutionary conservation of ACTN3 independent of ACTN2 suggests that other factors may modulate the expression of X allele and influence different aspects of variation in muscle function in older people. For example, animal-related studies have shown that restoration of α-actinin-3 does not increase muscle mass, but highlights the primary role of α-actinin-3 in modulating muscle metabolism with altered fatiguability (Garton et al., 2018). Additionally, studies suggest that there may be some male-specific functional declines in physical performance traits with the 557X allele (Delmonico et al., 2007; Ma et al., 2018). Ma et al. (2018) found that alternatively in women, ACTN3 577X alters structural and metabolic properties of the skeletal muscle, thus influencing frailty levels. Due to a larger proportion of women, the associations between the ACTN3 R577X genotype and metabolic properties of the skeletal muscle may be more pronounced in our population and contribute to the positive association of 577X allele with the sarcopenia.

The vitamin D receptor (VDR) is one of the candidate genes for sarcopenia, because it plays an important regulatory role in calcium homeostasis and skeletal muscle function (Whitfield et al., 2001). The VDR gene has several polymorphisms [restriction site polymorphisms *Apa*I (rs7975232), Cdx2 (rs11568820), *Bsm*I (rs1544410), *Fok*I (rs10735810) and *Taq*I (rs731236)] that have been previously investigated, but there is also controversy over the possible association between VDR genotypes and muscle mass and strength in older people. The VDR *Fok*I site is considered functional, resulting in two different VDR protein products (Arai et al., 1997; Whitfield et al., 2001): the VDR ff variant, which leads to a full length of the VDR protein, and the VDR FF variant, which leads to a shortened protein with three amino acids less (Whitfield et al., 2001). Some studies found no association between the VDR *Fok*I genotype and muscle mass (Moreno Lima et al., 2007; Bahat et al., 2010). In addition, Roth et al. (2004) reported that the VDR *Fok*I genotype was significantly associated with lean mass, but the results were not confirmed in their replication study (Walsh et al., 2016). On the other hand, there is evidence of higher quadriceps strength in ff compared to the FF carriers in older women (Windelinckx et al., 2007; Walsh et al., 2016) and older men (Roth et al., 2004; Windelinckx et al., 2007; Walsh et al., 2016). Despite the fact that VDR *Fok*I polymorphisms have been consistently associated with lower muscle strength, no study has confirmed the association with grip strength. Roth et al. (2004) and Walsh et al. (2016) also studied VDR polymorphisms in relation to sarcopenia. In one of the studies, they found a twofold higher risk of sarcopenia in men VDR *Fok*I FF homozygotes than in f allele carriers (Roth et al., 2004). In their replication study, the odds of sarcopenia in FF women were moderately higher compared to f allele carriers, but no significant differences in sarcopenia risk were observed in men (Walsh et al., 2016). Taken together, these results could indicate that genetic variation in VDR has a modest influence on lower muscle strength and risk of sarcopenia.

We could not detect any association of the VDR *Fok*I genotype with strength or sarcopenia in our cohort. A possible explanation could be the measurement of grip strength, which has been shown to lack f allele susceptibility. Moreover, given that VDR variation has a moderate effect on sarcopenia risk, a larger sample size of probably one hundred cases would be required to detect a significant association.

### Sarcopenia Genetic Risk Score

We tried to include genetic information for the purpose of risk prediction, based on a genetic risk score with multiple SNPs associated with sarcopenia. Since the contribution of a single gene polymorphism to the characteristics of sarcopenia is usually relatively small (<5%), it would be valuable to consider a calculation of a genetic risk score based on multiple genetic variants. Previous studies have already positively associated the polymorphisms in the genes ACTN3 (Cho et al., 2017), VDR (Roth et al., 2004; Walsh et al., 2016), and CVI (Lin et al., 2014) with sarcopenia, and it is likely that further common polymorphisms will be associated with sarcopenia in the future, opening up the possibility of developing a polygenic risk score approach to predict sarcopenia risk.

**FIGURE 3:**
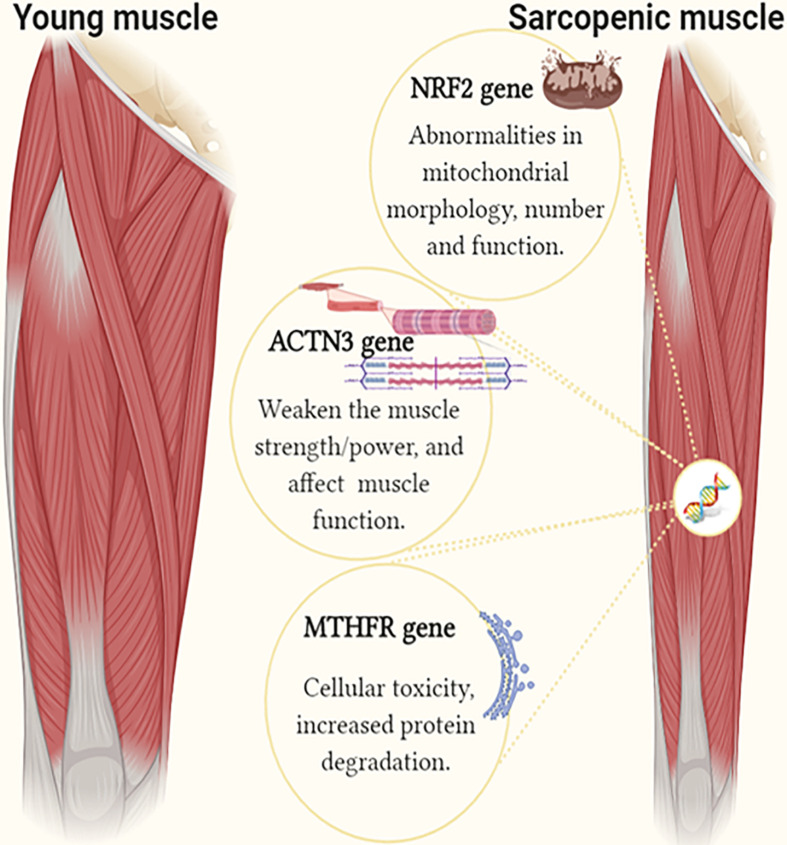
The image shows the identified genetic factors in individuals with sarcopenia compared to controls. The single and combined effect of MTHFR C (rs1801131 A/C), ACTN3 X (rs1815739 R/X), and NRF2 C (rs12594956 A/C) polymorphisms is associated with sarcopenia risk in older adults (Figure created using BioRender).

As shown in this study, the contribution of a single gene polymorphism ranged from 3.7 to 5%, while the combined effect of all three polymorphisms (MTHFR C, ACTN3 X, and NRF2 C) explained as much as 39% of the interindividual variability in sarcopenia risk. The combined effect of MTHFR C, ACTN3 X and NRF2 C alleles is likely to be detrimental to older adults, with three or more of these alleles increasing the likelihood of sarcopenia (Figure 3). The proportion of participants with at least one unfavorable allele was 1.06-fold higher in the sarcopenic participants compared to the controls. In addition, we calculated the total sarcopenia genetic risk score cut-off point, which maximizes the predictive value for the classification of sarcopenia. The cut-off point of ≥58.3% classifies subjects into the sarcopenia risk category.

### Study Limitations and Alternatives

The study was conducted with a relatively small sample size due to ineligibility criteria, such as advanced dementia, disease and non-response rate. The lack of statistical significance in some comparisons can therefore be misinterpreted as no difference between cases and controls. Due to the case-control nature of the study, we were also unable to explain the cause-effect relationship between the analyzed genotypes and sarcopenia. Therefore, large-scale longitudinal clinical studies would be useful to verify the findings and to gain further insights into the gene variants that contribute to sarcopenia. Furthermore, our data were not analyzed separately for men and women in this gene association study because of insufficient power to test the hypothesis. This means that we could have overlooked the sex-dependent gene association with sarcopenia. Different genes probably contribute to different aspects of muscle characteristics and may have different expressions in men and women. It will therefore be helpful to determine whether the association between MTHFR, NRF2, ACTN3 genes, and sarcopenia is sex-specific. The current findings need to be confirmed in a future study using a different population and a larger sample size to mitigate the potential bias.

We observed a deviation from the Hardy-Weinberg equilibrium in the sarcopenic samples (cases), but not in the control samples for the MTHFR 1.p36.2 A > C (rs1801131) polymorphism. Despite that, case-control studies require control samples to be in HWE, because a deviation from HWE in cases may indicate a genetic association. Even if the cases at a disease locus may be expected to show deviation from HWE under certain conditions, the association of the MTHFR gene with sarcopenia in our study should be considered with caution. Some errors, or other factors, could contribute to the observed deviation from HWE (Wittke-Thompson et al., 2005), and where our case is concerned, could be due to a small sample size or profound sex effects, rather than reflecting biological characteristics of the genetic disease model.

The study provides evidence that the genotypes of MTHFR, NRF2, and ACTN3 genes are associated with sarcopenia risk in older Caucasian adults. As far as we know, the associations of MTHFR and NRF2 variants with sarcopenia risk were shown for the first time. We would like to emphasize that the calculation of a genetic risk score can serve as a predictor of a higher risk of sarcopenia. This preliminary evidence, if confirmed in a larger cohort of samples, could be considered as a baseline for new insights into the pathogenesis and future diagnosis of sarcopenia.

## Data Availability Statement

The data that support the findings of this study are openly available in repository “Mendeley data” at http://dx.doi.org/10.17632/wh3c5z2htd.1.

## Ethics Statement

The studies involving human participants were reviewed and approved by the Republic of Slovenia National Medical Ethics Committee (0120-360-2018-10). The patients/participants provided their written informed consent to participate in this study.

## Author Contributions

All authors listed have made a substantial, direct and intellectual contribution to the work, and approved it for publication.

## Conflict of Interest

The authors declare that the research was conducted in the absence of any commercial or financial relationships that could be construed as a potential conflict of interest.

## Footnotes

1 https://www.ncbi.nlm.nih.gov/snp

2 snpedia.com

3 phgkb.cdc.gov/PHGKB/phenoPedia

4 https://zzz.bwh.harvard.edu/gpc/#cc_ins
