# Supplementary material for: Pilot Study on Genetic Associations With Age-Related Sarcopenia
Source: Front Genet. 2021 Jan 11;11:615238. doi: 10.3389/fgene.2020.615238 (PMC7831746; doi:10.3389/fgene.2020.615238)
Supplement: Supplementary file 1 [file Table_1.DOCX]

Supplementary Material

# Supplementary Tables

**Table S1.** Genotypes and alleles count of MTHFR, ACTN3, NRF2, ADRB2, VDR, and NPAS4 related to sarcopenia in the study population

|  | **Genotype frequency** | | | |  | **Alleles frequency** | | | | |
| --- | --- | --- | --- | --- | --- | --- | --- | --- | --- | --- |
|  |  | **No-sarcopenia** | **Sarcopenia** |  |  |  | **No-sarcopenia** | **Sarcopenia** |  |  |
|  |  | ***n* (%)** | ***n* (%)** | ***p*** |  |  | ***n* (%)** | ***n* (%)** | ***p*** | ***OR* (95% CI)** |
| MTHFR (rs1801131) | AA | 72 (49.7) | 13 (28.9) | <.001 |  | A | 208 (71.7) | 41 (45.6) | <.001 | 3.032 (1.862–4.935) |
|  | AC | 64 (44.1) | 15 (33.3) |  |  | C | 82 (28.3) | 49 (54.4) |  |  |
|  | CC | 9 (6.2) | 17 (37.8) |  |  |  |  |  |  |  |
| ACTN3  (rs1815739) | RR | 46 (31.7) | 8 (17.8) | .043 |  | R | 167 (57.6) | 36 (40.0) | .003 | 2.037 (1.258–3.297) |
|  | RX | 71 (49.0) | 21 (46.7) |  |  | X | 123 (42.4) | 54 (60.0) |  |  |
|  | XX | 28 (19.3) | 16 (35.5) |  |  |  |  |  |  |  |
| NRF2  (rs12594956) | AA | 35 (24.1) | 4 (8.9) | .015 |  | A | 149 (51.4) | 32 (35.6) | .009 | 1.915 (1.174–3.124) |
|  | AC | 81 (55.9) | 24 (53.3) |  |  | C | 141 (48.6) | 58 (64.4) |  |  |
|  | CC | 29 (20.0) | 17 (38.6) |  |  |  |  |  |  |  |
| ADRB2 (rs1042713) | AA | 30 (20.7) | 4 (8.9) | .193 |  | A | 118 (40.7) | 28 (32.1) | .103 | 1.519 (0.918–2.515) |
|  | AG | 58 (40.0) | 20 (44.4) |  |  | G | 172 (59.3) | 62 (67.9) |  |  |
|  | GG | 57 (39.3) | 21 (46.7) |  |  |  |  |  |  |  |
| VDR (rs2228570) | CC | 53 (36.6) | 18 (40.0) | .645 |  | C | 181 (62.4) | 56 (62.2) | .974 | 1.008 (0.619–1.642) |
|  | CT | 75 (51.7) | 20 (44.4) |  |  | T | 109 (37.6) | 34 (37.8) |  |  |
|  | TT | 17 (11.7) | 7 (15.6) |  |  |  |  |  |  |  |
| NPAS4 (rs7947391) | AA | 16 (11.0) | 3 (6.7) | .652 |  | A | 111 (38.3) | 33 (36.7) | .783 | 1.071 (0.656–1.748) |
|  | AG | 79 (54.5) | 27 (60.0) |  |  | G | 179 (61.7) | 57 (63.3) |  |  |
|  | GG | 50 (34.5) | 15 (33.3) |  |  |  |  |  |  |  |

**Table S2.** Physical parameters according to MTHFR, ACTN3, NRF2, ADRB2, VDR, CX3CR1, NPAS4 genotypes

|  | **Gender** | **Genotype (*n* = men/women)** | | | ***p* - values** |
| --- | --- | --- | --- | --- | --- |
|  |  | MTHFR | | |  |
|  |  | AA (n = 27/63) | AC (n = 27/45) | CC (n = 13/15) |  |
| Skeletal mass index (kg/m^2^) | Men | 8.27 (1.19) | 8.32 (1.11) | 7.85 (1.09) | .456 |
|  | Women | 6.74 (0.88) | 6.76 (0.79) | 6.09 (0.73)^*^**^,**^** | .019 |
| Grip strength (kg) | Men | 23.3 (7.99) | 30.1 (11.2) | 24.85 (12.6)^**^ | .052 |
|  | Women | 17.3 (6.18) | 16.7 (6.50) | 12.37 (5.87)**^*,**^** | .024 |
| Gait speed (m/s) | Men | 0.67 (0.33) | 0.81 (0.42) | 0.71 (0.38) | .360 |
|  | Women | 0.81 (0.44) | 0.68 (0.37) | 0.65 (0.39) | .164 |
|  |  | ACTN3 | | |  |
|  |  | RR (n = 19/35) | RX (n = 35/57) | XX (n = 13/31) |  |
| Skeletal mass index (kg/m^2^) | Men | 8.27 (1.08) | 8.24 (1.11) | 8.04 (1.35) | .846 |
|  | Women | 6.85 (1.04) | 6.58 (0.75) | 6.63 (0.79) | .333 |
| Grip strength (kg) | Men | 25.8 (8.52) | 28.8 (11.9) | 20.5 (7.62) | .054 |
|  | Women | 17.8 (6.48) | 16.4 (5.90) | 15.0 (7.07) | .199 |
| Gait speed (m/s) | Men | 0.79 (0.40) | 0.73 (0.39) | 0.65 (0.32) | .582 |
|  | Women | 0.81 (0.44) | 0.76 (0.41) | 0.38 (0.64) | .209 |
|  |  | NRF2 | | |  |
|  |  | AA (n = 9/30) | AC (n = 41/64) | CC (n = 17/29) |  |
| Skeletal mass index (kg/m^2^) | Men | 8.74 (0.82) | 8.18 (1.18) | 8.02 (1.14) | .292 |
|  | Women | 6.77 (0.85) | 6.67 (0.75) | 6.58 (1.07) | .700 |
| Grip strength (kg) | Men | 35.4 (14.34) | 25.6 (9.67) | 23.3 (8.68) **^*,**^** | .015 |
|  | Women | 17.6 (6.13) | 17.1 (6.51) | 14.0 (6.03) | .058 |
| Gait speed (m/s) | Men | 0.95 (0.32) | 0.77 (0.39) | 0.55 (0.29)**^*^** | .024 |
|  | Women | 0.81 (0.43) | 0.76 (0.41) | 0.64 (0.40) | .294 |
|  |  | ADRB2 | | |  |
|  |  | AA (n = 8/26) | AG (n = 31/47) | GG (n = 28/50) |  |
| Skeletal mass index (kg/m^2^) | Men | 8.20 (1.08) | 8.13 (1.19) | 8.31 (1.13) | .828 |
|  | Women | 6.84 (0.75) | 6.53 (0.86) | 6.71 (0.89) | .327 |
| Grip strength (kg) | Men | 22.0 (8.31) | 25.4 (10.4) | 26.9 (12.9) | .547 |
|  | Women | 19.1 (6.35) | 15.8 (5.73) | 15.7 (6.79) | .056 |
| Gait speed (m/s) | Men | 0.71 (0.26) | 0.72 (0.42) | 0.75 (0.37) | .936 |
|  | Women | 0.81 (0.46) | 0.78 (0.40) | 0.68 (0.39) | .312 |
|  |  | VDR | | |  |
|  |  | CC (n = 22/49) | CT (n = 35/59) | TT (n = 8/15) |  |
| Skeletal mass index (kg/m^2^) | Men | 8.46 (1.24) | 8.18 (1.173) | 8.16 (1.08) | .794 |
|  | Women | 6.52 (0.78) | 6.83 (0.97) | 6.51 (0.69) | .116 |
| Grip strength (kg) | Men | 28.6 (12.0) | 24.1 (9.88) | 29.5 (9.23) | .187 |
|  | Women | 16.2 (5.39) | 17.5 (7.07) | 13.3 (5.96) | .070 |
| Gait speed (m/s) | Men | 0.78 (0.45) | 0.69 (0.31) | 0.84 (0.47) | .480 |
|  | Women | 0.75 (0.42) | 0.77 (0.43) | 0.65 (0.35) | .590 |
|  |  | NPAS4 | | |  |
|  |  | AA (n = 5/14) | AG (n = 41/65) | GG (n = 21/44) |  |
| Skeletal mass index (kg/m^2^) | Men | 8.15 (1.31) | 8.20 (1.16) | 8.24 (1.12) | .985 |
|  | Women | 6.59 (0.92) | 6.54 (0.87) | 6.89 (0. 68) | .111 |
| Grip strength (kg) | Men | 25.8 (5.72) | 26.6 (11.7) | 25.9 (9.79) | .962 |
|  | Women | 15.1(4.04) | 16.2 (6.10) | 17.3 (7.40) | .462 |
| Gait speed (m/s) | Men | 0.58 (0.28) | 0.71 (0.36) | 0.83 (0.43) | .314 |
|  | Women | 0.62 (0.32) | 0.77 (0.43) | 0.76 (0.42) | .451 |

*Note*. * denotes that aa homozygotes were scientifically different compared to AA homozygotes, and ** denotes that aa homozygotes were significantly different compared to Aa heterozigotes.

**Table S3. Coordinates of the ROC curve for Sarcopenia Genetic Risk Score cut-off point**

| **Positive if greater than or equal to** | **Sensitivity** | **1 - Specificity** |
| --- | --- | --- |
| 58.333 | 0.568 | 0.151 |

**Table S4. Area under the ROC curve for the Sarcopenia Genetic Risk Score**

| **Area** | **p-value** | **95% Confidence Interval** | |
| --- | --- | --- | --- |
| 0.774 | 0.000 | 0.695 | 0.853 |
